# Could surgical transepicondylar axis be identified accurately in preoperative 3D planning for total knee arthroplasty? A reproducibility study based on 3D-CT

**DOI:** 10.1186/s42836-022-00147-2

**Published:** 2022-10-17

**Authors:** Kai Lei, Li Ming Liu, Jiang Ming Luo, Chao Ma, Qing Feng, Liu Yang, Lin Guo

**Affiliations:** 1 Center for Joint Surgery, Southwest Hospital, Third Military Medical University, No. 30 Gaotanyan Street, Shapingba District, Chongqing, 400038 China; 2 Minimally Invasive Gastrointestinal Surgery Center, Southwest Hospital, Third Military Medical University, No. 30 Gaotanyan Street, Shapingba District, Chongqing, 400038 China

**Keywords:** Total knee arthroplasty, Femoral component rotation, Transepicondylar axis, Reproducibility, Three-dimensional

## Abstract

**Background:**

Surgical transepicondylar axis (sTEA) is frequently used for positioning of femoral component rotation in total knee arthroplasty (TKA). Previous studies showed that intraoperative identification of sTEA was not reliable. While surgeons or engineers need to identify sTEA with three-dimensional (3D) computer-aid techniques pre- or intraoperatively, the reproducibility of sTEA identification on preoperative 3D images has not been explored yet. This study aimed to investigate the reproducibility of identifying sTEA in preoperative planning based on computed tomography (CT).

**Methods:**

Fifty-nine consecutive patients (60 knees involved) who received TKA in our center from April 2019 to June 2019 were included in this study. Six experienced TKA surgeons identified sTEA three times on 3D model established on the basis of knee CT data. The projection angle of each sTEA and the posterior condyle axis on the transverse plane were measured and analyzed.

**Results:**

The overall intra-observer reproducibility was moderate. The median intra-observer variation was 1.27°, with a maximum being up to 14.07°. The median inter-observer variation was 1.24°, and the maximum was 11.47°. The overall intra-class correlation coefficient (ICC) for inter-observer was 0.528 (95% CI 0.417, 0.643).

**Conclusion:**

The identification of sTEA on a 3D model established on the basis of knee CT data may not be reliable. Combined with the previous cadaveric and surgical studies, caution should be exercised in determining femoral component rotation by referencing sTEA both preoperatively and intraoperatively.

**Level of evidence:**

III

**Supplementary Information:**

The online version contains supplementary material available at 10.1186/s42836-022-00147-2.

## Introduction

Total knee arthroplasty (TKA) is a successful procedure to manage late-stage knee osteoarthritis. Multiple factors may influence the clinical results of TKA. Equal extension and flexion gap is one of the key objectives of TKA to achieve satisfactory soft tissue balance. Femoral component malrotation could be one of the major causes of unbalanced flexion gap. It may cause pain, knee stiffness, patellofemoral mal-tracking and reduced implant longevity after TKA [1–7]. The references for femoral component rotation include: posterior condyle axis [8], Whiteside's line [9], transepicondylar axis [10], sulcus line [11], and tibia osteotomy platform [12]. Among all the commonly used references for measured resection technique, surgical transepicondylar axis (sTEA) is deemed the best flexion-extension axis of knee joint [10, 13–17], starting from the most prominent point of the lateral femoral epicondyle to the most concave point of the medial femoral epicondyle [10, 14].

However, it is somehow difficult to accurately identify sTEA during operation by palpating bony prominences, due to poor vision, soft tissue coverage, obscure bony landmarks, *etc*. [18–21]. The individual differences between operators in locating sTEA are obvious [18–20, 22–24]. In order to achieve better femoral component rotation and flexion gap, more precise measurement is needed to locate femoral rotation axis based on sTEA methodology. With wider application of navigation, patient-specific instrumentation (PSI), robotics or other computer-assisted surgical techniques [25–28], sTEA could be identified by surgeons or engineers during preoperative planning with assistance of three-dimensional (3D) images. Intraoperative femoral rotation osteotomy could be guided by robot, PSI or navigation after identification of sTEA preoperatively or intraoperatively [29]. Even without these techniques, the divergence between sTEA and posterior condylar axis could be measured on a preoperative 3D model based on computed tomography (CT) data and serve to orientate sTEA as a reference during surgery [27].

However, identifying sTEA on a 3D model does not go further than defining sTEA by recognizing bony prominences. Although most soft tissue signals were removed from 3D CT images, the prominence, as a landmark, may not be reproducible to draw a unique sTEA. The purpose of this study was to investigate the intra- and inter-observer reproducibility for identification of the sTEA on 3D images. Previous studies on reproducibility of sTEA were carried out on cadeveric specimens or patients' knees [18–20, 22–24]. To our knowledge, this was the first attempt to explore the accuracy and reproducibility of sTEA measurement on 3D images. We hypothesized that the reproducibility of sTEA identification on 3D image remains too poor to make 3D sTEA a reliable landmark for attaining precise femoral rotation and flexion gap.

## Materials and methods

Preoperative femoral CT data were retrospectively collected from a series of consecutive TKAs at our center from April 2019 to June 2019. Patients with any bony deformities that affected recognition of femoral bony prominence and sTEA positioning were excluded, such as previous fractures, severe osteophytes and any extra-articular deformities. Finally, 59 consecutive patients involving 60 knees were included in the study. There were 9 males and 50 females, with 29 left and 31 right knees included. Mean age at the time of surgery was 67.7 years ± 8.2 years (range, 50 to 86). Mean height was 156.9 cm ± 6.2 cm (range, 145 to 172) and mean weight was 62.8 Kg ± 9.9 Kg (range, 40 to 90), with a mean body mass index of 25.5 Kg/m^2^ ± 3.9 Kg/m^2^ (range, 16.4 to 35.2).

**Fig. 1:**
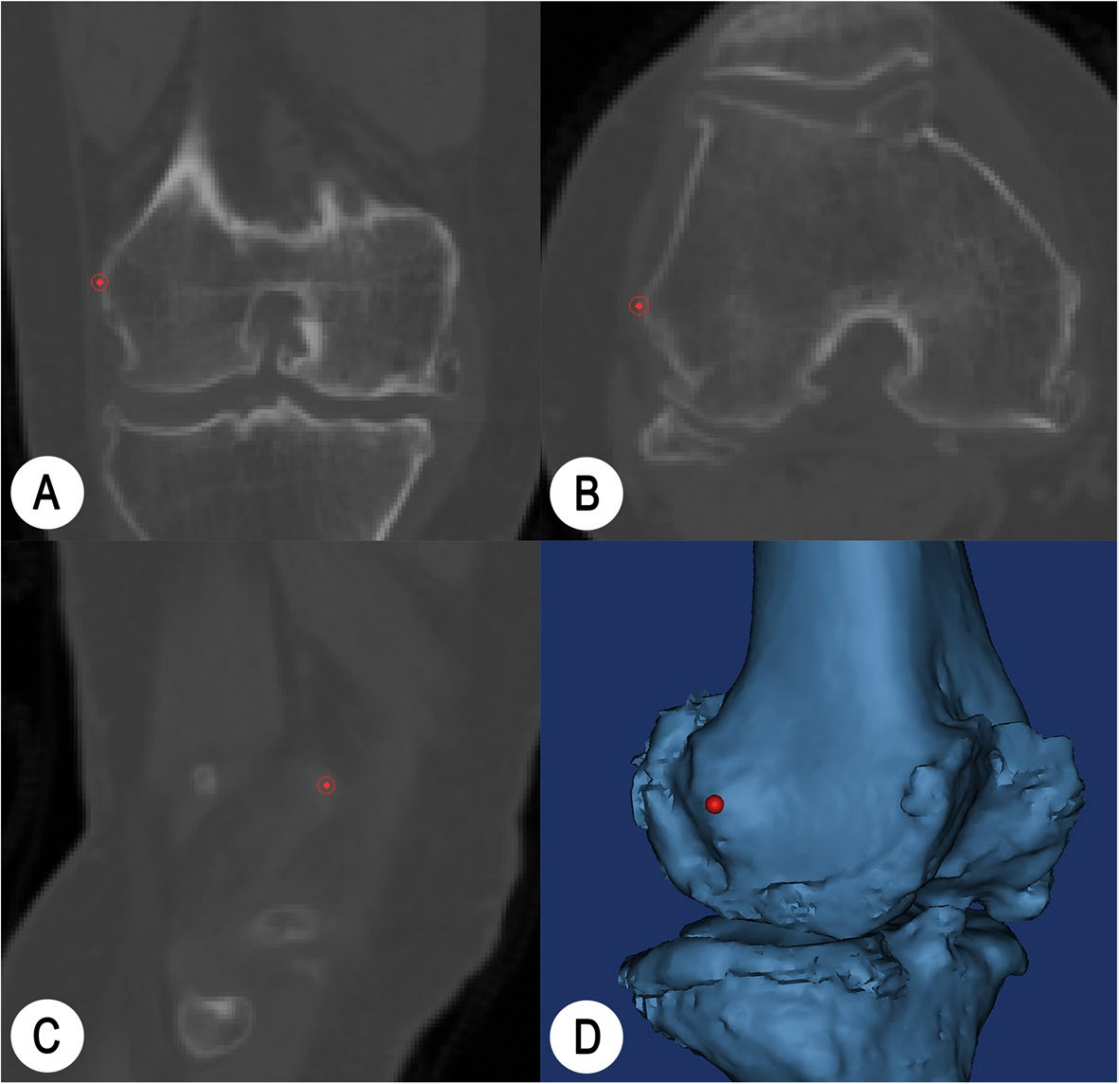
**A**-**D** are images shown in Mimics Research 19.0 that combine the coronal, axial, sagittal, and 3D views to mark the most prominent point of the lateral femoral condyle, respectively

A 3D reconstruction was conducted on the basis of preoperative femoral CT data (thinner scan of 1 mm around knee and thicker scan of 3 mm for the rest parts) by employing Mimics Research 19.0 (Materialise NV, Belgium). The best spherical fitting of femoral head was performed with CATIA 5.20 (Dassault System, France), and the line between the obtained center point and the apex of femoral intercondylar notch was defined as the mechanical axis of femur; the plane perpendicular to the femoral mechanical axis was recorded as the transverse plane; the tangent line to the most posterior part of the femoral condyles was defined as the posterior condyle axis [17, 27]. Six experienced TKA surgeons identified sTEA three times independently on axial, coronal, sagittal, and 3D views [27, 30] (Fig. 1), with an interval of more than 15 days. Each identification was based on the initial model to ensure that there was no interference from other marker information.

**Fig. 2:**
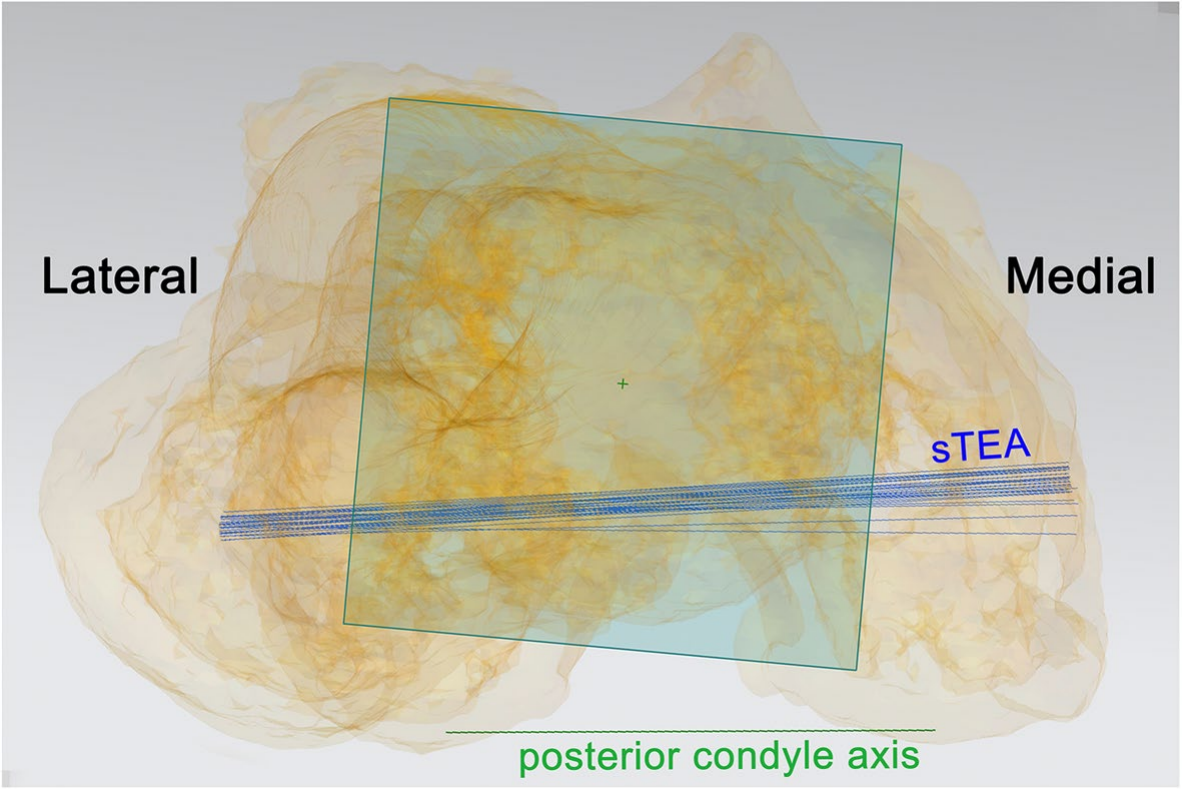
Eighteen surgical transepicondylar axes and one posterior condyle axis are marked on 3D knee model

**Fig. 3:**
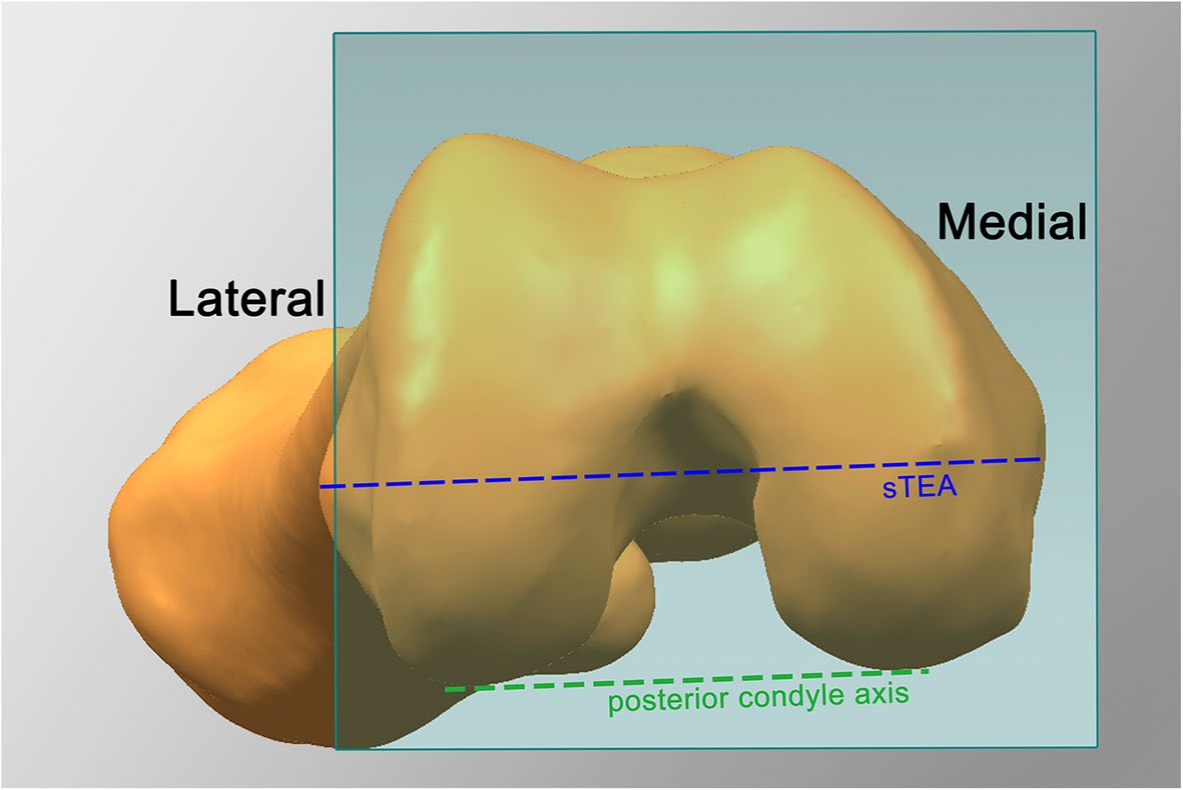
The blue plane represents the transverse plain; the blue and green dashed line are the projections of sTEA and posterior condyle axis on transverse plain, respectively. The angle between the above two lines is denoted as posterior condylar angle (PCA)

A total of 18 sTEA were marked for each 3D model of the knee joint (Fig. 2). Unigraphics NX 12.0 (Siemens PLM Software, USA) was used to measure projection angle of each sTEA and its corresponding posterior condyle axis on the transverse plane, which was denoted as posterior condylar angle (PCA). Since there was only one fixed posterior condyle axis in each model, the variation of PCA was only related to precision errors on the sTEA. Relative to the posterior condyle axis, sTEA external rotation would record PCA as positive and internal negative (Fig. 3).

To evaluate intra-observer reproducibility, intra-class correlation coefficients (ICC) and their 95% confident intervals (CI) were calculated based on a two-way mixed, absolute agreement, single-measure model. For inter-observer reproducibility, two-way random, absolute agreement, single-measure model was utilized [31]. And ICC values less than 0.5, between 0.5 and 0.75, between 0.75 and 0.9, and greater than 0.90 were indicative of poor, moderate, good and excellent reproducibility, respectively [31]. Variables with normal distribution were expressed as $\overline{x}\pm s$, variables with high skew were presented as median and interquartile range (IQR). Statistical analyses were performed by using SPSS 25.0 (SPSS Inc, Chicago, IL).

This study has been approved by the local Ethics Committee (KY2020057).

## Results

Intra-observer ICCs were 0.720, 0.516, 0.652, 0.717, 0.548, 0.503, respectively. Overall intra-observer reproducibility was moderate, standing somewhere between 0.5 and 0.75. Median (IQR) of overall intra-surgeon variation was 1.27° (0.52°, 2.41°), with a maximum of up to 14.07° (Table 1). Median (IQR) of inter-surgeon variation was 1.24° (0.55°, 2.37°), and the maximum was 11.47°. Overall ICC for inter-surgeon was 0.528 (95%CI 0.417, 0.643) (Table 2). Descriptive statistics of PCA for each knee model are given in the appendix (Appendix 1).

**Table 1:** Intra-observer reproducibility and variation

Intra-observer	Reproducibility		Variation (degrees)	
	ICC	95% CI	Median (IQR)	Range (min, max)
Observer A	0.720	0.609, 0.811	1.172 (0.467, 2.149)	0.002, 6.323
Observer B	0.516	0.368, 0.653	1.081 (0.477, 2.200)	0.001, 14.070
Observer C	0.652	0.525, 0.761	1.146 (0.475, 1.957)	0.006, 13.143
Observer D	0.717	0.605, 0.809	0.744 (0.278, 1.717)	0.006, 9.172
Observer E	0.548	0.401, 0.681	1.779 (0.865, 3.661)	0.006, 11.560
Observer F	0.503	0.338, 0.649	1.624 (0.851, 2.933)	0.018, 8.711

*ICC* intraclass correlation efficient, *CI* confidence interval, *IQR* interquartile range.

**Table 2:** Inter-observer reproducibility and variation

Inter-observer		Reproducibility		Variation (degrees)	
		ICC	95% CI	Median (IQR)	Range (min, max)
Observer A	Observer B	0.797	0.683, 0.874	0.910 (0.425, 1.561)	0.013, 4.455
	Observer C	0.642	0.464, 0.770	1.209 (0.644, 2.069)	0.004, 9.816
	Observer D	0.763	0.634, 0.851	0.854 (0.463, 1.430)	0.043, 7.199
	Observer E	0.553	0.297, 0.724	1.541 (0.563, 2.717)	0.018, 9.187
	Observer F	0.562	0.360, 0.713	1.302 (0.573, 2.445)	0.025, 5.158
Observer B	Observer C	0.615	0.431, 0.750	0.920 (0.446, 1.756)	0.002, 9.828
	Observer D	0.730	0.585, 0.830	1.052 (0.475, 1.632)	0.026, 5.424
	Observer E	0.538	0.317, 0.700	1.773 (0.758, 3.253)	0.016, 6.868
	Observer F	0.410	0.179, 0.599	1.731 (0.532, 2.844)	0.007, 6.427
Observer C	Observer D	0.568	0.369, 0.717	0.921 (0.519, 1.662)	0.044, 8.495
	Observer E	0.217	-0.017, 0.435	2.371 (1.006, 3.944)	0.027, 11.466
	Observer F	0.485	0.263, 0.657	1.402 (0.529, 2.719)	0.059, 6.910
Observer D	Observer E	0.574	0.353, 0.729	1.785 (0.626, 2.661)	0.013, 6.824
	Observer F	0.405	0.172, 0.596	1.353 (0.373, 2.298)	0.026, 10.660
Observer E	Observer F	0.278	0.041, 0.490	2.078 (0.687, 3.787)	0.147, 9.961

*ICC* intraclass correlation efficient, *CI* confidence interval, *IQR* interquartile range.

## Discussion

The application of computer-assisted techniques in TKA is growing as the importance of bone cut precision in gap-balancing has been increasingly recognized [32]. When a surgeon uses measured resection technique to perform TKA with the assistance of image-based navigation, PSI or robotics, the determination of femoral component rotation plan is relied on the identification of sTEA on the basis of preoperative imaging data (CT or Magnetic Resonance Imaging) [25–28]. However, previous studies on reproducibility of sTEA were carried out on cadaveric knees or patients’ knees during TKA [18–20, 22–24]. Stoeckl *et al*. marked sTEA on cadaveric knees multiple times and acquired a nearly 3 cm^2^ area on both medial and lateral condyles [23]. Jerosch, Yan and Siston yielded comparable results on cadavers [19, 22, 24]. Jenny *et al*. identified sTEA on patients' knees during TKA and evaluated the results with navigation. They found that intra-observer deviation was 5° to 6° with a maximum deviation of 15° and the inter-observer deviation was about 9° with a maximum deviation of 15° [18]. Kinzel’s study also arrived at similar conclusion [20]. All these studies came to the same conclusion that identification of sTEA is not reliable on bony prominences. Although the 3D color mapping proposed by Xiang *et al*. might be a feasible alternative for locating sTEA, it still warrants further clinical verification [33]. Reproducibility of sTEA is poor as a routine reference for TKA regardless of whether sTEA is the true center for femoral rotation.

This study focused, for the first time, on the preoperative reproducibility of sTEA identified on the basis of 3D CT data. It was found that when the same observer identified sTEA at different times, the median variation was 1.27°, with a maximum variation of up to 14.07°. Even if surgeons repeatedly identified sTEA and calculated the average, the median variation among different surgeons was 1.24°, and the maximum was 11.47°. This finding indicated that reproducibility of sTEA based on knee CT data was only moderate in preoperative planning. Many studies showed that PSI could not improve the rotational alignment of femoral implant compared with conventional methods [21, 34–36]. And similar conclusions could be drawn for navigation and robotic surgeries [37–41]. The poor reproducibility of sTEA in preoperative or intraoperative 3D planning may be one of the main reasons for the aforementioned phenomenon.

This study also indicated that identifiability of bony anatomical landmarks was positively correlated with the reproducibility of sTEA identification (Appendix 1). Besides, there may exist a possible positive correlation between intra-observers' reproducibility and their experience in TKA, which is worth further study. Only in patients with clear anatomical bony prominence on medial and lateral condyles, techniques, such as image-based navigation, PSI, robotics or personalized 3D preoperative planning, could reproduce the femoral component rotation axis. And the accuracy of sTEA identification relies on a surgeon’s experience. Surgeons should be more cautious when using sTEA before and during TKA. It may be more reliable to refer to multiple reference axes or use gap balancing technique to get the appropriate femoral rotation and flexion gap.

This study has multiple limitations. First, the number of observers and cases for identifying sTEA were limited. Second, this study was a single-center one, and its reproducibility and execution need to be further verified in other centers. Third, further researches are needed to establish the relationship of sTEA reproducibility with bony prominence identifiability and observer experience.

## Conclusion

The identification of sTEA on 3D model established on the basis of knee CT data in preoperative planning may not be reliable. Combined with the previous cadaveric and surgical studies, caution should be exercised in determining femoral component rotation by referencing sTEA both preoperatively and intraoperatively.

## Supplementary Information

**Additional file 1.** Descriptive statistics of PCA for each knee model. The above four dynamic graphics (if dynamic graphics are not moving, please check out videos in Appendix 2, 3, 4 and 5) showed 3D knee model of No. 6, 15, 29 and 56, respectively. It could be found that bony anatomical landmarks of No. 6 and No. 15 were obscurer than those of No. 29 and No. 56, especially in the medial femoral epicondyle, which indicates that identifiability of bony anatomical landmarks is positively correlated with the reproducibility of identifying sTEA.

**Additional file 2.**

**Additional file 3.**

**Additional file 4.**

**Additional file 5.**

## Data Availability

All data and materials are available on reasonable request.

## Abbreviations

TKA: Total knee arthroplasty
sTEA: Surgical transepicondylar axis
PSI: Patient specific instrumentation
3D: Three-dimensional
CT: Computed tomography
PCA: Posterior condylar angle
ICC: Intraclass correlation efficient
CI: Confidence interval
IQR: Interquartile range
