# Supplementary material for: Could surgical transepicondylar axis be identified accurately in preoperative 3D planning for total knee arthroplasty? A reproducibility study based on 3D-CT
Source: Arthroplasty. 2022 Oct 17;4:46. doi: 10.1186/s42836-022-00147-2 (PMC9575283; doi:10.1186/s42836-022-00147-2)
Supplement: Supplementary file 1 — Additional file 1. Descriptive statistics of PCA for each knee model. The above four dynamic graphics (if dynamic graphics are not moving, please check out videos in Appendix 2, 3, 4 and 5) showed 3D knee model of No. 6, 15, 29 and 56, respectively. It could be found that bony anatomical landmarks of No. 6 and No. 15 were obscurer than those of No. 29 and No. 56, especially in the medial femoral epicondyle, which indicates that identifiability of bony anatomical landmarks is positively correlated with the reproducibility of identifying sTEA. [file 42836_2022_147_MOESM1_ESM.docx]

**Appendix 1**

***Descriptive statistics of PCA for each knee model.***

|  | Min | P_10_ | P_25_ | P_50_ | P_75_ | P_90_ | Max | Range |
| --- | --- | --- | --- | --- | --- | --- | --- | --- |
| 1 | 1.17 | 1.22 | 1.60 | 2.78 | 3.46 | 4.58 | 5.13 | 3.96 |
| 2 | -9.28 | -5.93 | -3.70 | -2.89 | -2.57 | -1.87 | -1.10 | 8.18 |
| 3 | -0.55 | 2.76 | 4.79 | 6.91 | 8.25 | 10.94 | 11.29 | 11.84 |
| 4 | -4.41 | -2.66 | -1.89 | -1.40 | -0.69 | -0.18 | 0.33 | 4.74 |
| 5 | -0.83 | 4.21 | 5.07 | 7.00 | 8.55 | 9.75 | 11.22 | 12.05 |
| 6 | -1.09 | -0.67 | 0.10 | 1.56 | 4.21 | 13.03 | 14.79 | 15.89 |
| 7 | -2.85 | -1.88 | -1.44 | -1.12 | -0.19 | 1.84 | 2.63 | 5.48 |
| 8 | -2.05 | -1.91 | -1.04 | 0.39 | 0.98 | 2.21 | 7.17 | 9.22 |
| 9 | -1.35 | -0.73 | -0.05 | 1.02 | 3.87 | 6.09 | 6.52 | 7.88 |
| 10 | 1.97 | 2.99 | 3.55 | 4.14 | 6.03 | 7.53 | 7.73 | 5.76 |
| 11 | -2.16 | -1.39 | -0.90 | 0.11 | 1.49 | 3.52 | 3.60 | 5.76 |
| 12 | -3.24 | -1.04 | 0.75 | 1.12 | 3.24 | 5.23 | 5.68 | 8.92 |
| 13 | -3.82 | -0.60 | 1.38 | 2.33 | 3.08 | 4.24 | 6.61 | 10.44 |
| 14 | -1.50 | -1.49 | 1.02 | 2.90 | 6.64 | 7.30 | 7.49 | 8.99 |
| 15 | -4.00 | -0.45 | 0.51 | 4.50 | 8.83 | 10.43 | 12.87 | 16.88 |
| 16 | -2.32 | -2.04 | -0.40 | 0.22 | 0.89 | 2.59 | 3.47 | 5.79 |
| 17 | -0.33 | 0.08 | 1.04 | 2.09 | 2.55 | 3.82 | 7.17 | 7.51 |
| 18 | -0.11 | -0.01 | 0.99 | 2.13 | 3.65 | 4.16 | 5.12 | 5.23 |
| 19 | -0.32 | 0.08 | 0.77 | 1.18 | 3.70 | 4.56 | 6.43 | 6.74 |
| 20 | 0.85 | 1.17 | 1.79 | 3.22 | 3.63 | 4.60 | 7.70 | 6.85 |
| 21 | -4.05 | -2.33 | 0.28 | 1.51 | 2.26 | 3.48 | 5.32 | 9.37 |
| 22 | -6.16 | -4.13 | -2.22 | -0.96 | 0.15 | 2.53 | 5.40 | 11.56 |
| 23 | -0.29 | 0.11 | 2.13 | 3.09 | 3.71 | 4.78 | 5.29 | 5.57 |
| 24 | -0.58 | 0.48 | 1.76 | 2.73 | 3.23 | 3.93 | 3.97 | 4.55 |
| 25 | 0.35 | 0.57 | 2.29 | 3.01 | 4.04 | 6.10 | 12.83 | 12.48 |
| 26 | -4.53 | -4.00 | -3.38 | -1.25 | 2.31 | 4.26 | 5.33 | 9.86 |
| 27 | -1.02 | -0.58 | -0.06 | 0.50 | 0.84 | 1.42 | 3.31 | 4.33 |
| 28 | -5.06 | -4.20 | -3.49 | -0.03 | 3.82 | 5.44 | 9.72 | 14.77 |
| 29 | 2.65 | 2.82 | 3.54 | 4.32 | 4.47 | 4.90 | 5.28 | 2.64 |
| 30 | -1.22 | -0.51 | 1.27 | 3.23 | 4.41 | 5.30 | 5.62 | 6.84 |
| 31 | -1.05 | -0.73 | 0.17 | 0.77 | 1.74 | 3.62 | 6.93 | 7.98 |
| 32 | 3.31 | 3.91 | 4.42 | 4.80 | 5.38 | 6.04 | 7.07 | 3.76 |
| 33 | -3.49 | -2.61 | -1.69 | -0.34 | 0.14 | 1.29 | 4.14 | 7.63 |
| 34 | -4.03 | -0.37 | 2.65 | 3.22 | 4.56 | 5.43 | 7.67 | 11.70 |
| 35 | -2.33 | -1.80 | 0.04 | 1.78 | 8.18 | 10.08 | 10.86 | 13.19 |
| 36 | -5.19 | -2.86 | -1.51 | -0.29 | 0.36 | 1.64 | 4.10 | 9.29 |
|  |  |  |  |  |  |  |  | (Continued) |
| (Continued) | |  |  |  |  |  |  |  |
|  | Min | P_10_ | P_25_ | P_50_ | P_75_ | P_90_ | Max | Range |
| 37 | -0.55 | -0.38 | 1.41 | 2.77 | 4.10 | 8.85 | 12.32 | 12.87 |
| 38 | -3.31 | -1.14 | 1.17 | 1.84 | 2.30 | 3.90 | 6.81 | 10.13 |
| 39 | -3.88 | -3.58 | -2.33 | 0.08 | 1.07 | 1.70 | 1.76 | 5.63 |
| 40 | -3.08 | -0.77 | -0.26 | 0.79 | 3.36 | 5.88 | 6.38 | 9.46 |
| 41 | -3.33 | -2.74 | -1.86 | -0.31 | 4.41 | 6.32 | 10.74 | 14.07 |
| 42 | -4.10 | -3.86 | -0.02 | 1.37 | 2.60 | 3.33 | 3.50 | 7.59 |
| 43 | -0.27 | -0.17 | 0.35 | 1.47 | 2.66 | 7.03 | 8.10 | 8.37 |
| 44 | -7.13 | -3.60 | -1.83 | -0.72 | 0.51 | 3.40 | 9.51 | 16.64 |
| 45 | -0.21 | 1.16 | 2.07 | 3.07 | 3.79 | 4.57 | 5.36 | 5.57 |
| 46 | 1.26 | 1.55 | 2.17 | 2.64 | 3.27 | 4.38 | 5.47 | 4.21 |
| 47 | -2.72 | -1.33 | 1.71 | 3.04 | 4.31 | 6.01 | 6.66 | 9.38 |
| 48 | -2.52 | -1.47 | 0.08 | 0.84 | 1.93 | 2.20 | 2.28 | 4.80 |
| 49 | -5.07 | -4.21 | -1.30 | 1.51 | 3.23 | 3.79 | 5.13 | 10.21 |
| 50 | -1.53 | -1.28 | 1.03 | 2.42 | 3.65 | 4.99 | 5.70 | 7.24 |
| 51 | -4.35 | -4.35 | -3.72 | -1.95 | -0.86 | -0.44 | 0.27 | 4.62 |
| 52 | 0.78 | 1.25 | 2.24 | 2.81 | 3.95 | 5.69 | 5.85 | 5.07 |
| 53 | 1.50 | 1.80 | 3.27 | 4.35 | 6.11 | 7.80 | 8.37 | 6.88 |
| 54 | -2.42 | -1.70 | 0.27 | 0.99 | 1.76 | 2.31 | 2.44 | 4.86 |
| 55 | -0.96 | -0.77 | 1.94 | 3.20 | 3.87 | 4.24 | 5.11 | 6.07 |
| 56 | 2.56 | 2.60 | 2.72 | 3.44 | 4.10 | 4.32 | 4.58 | 2.01 |
| 57 | -1.60 | -1.47 | -0.49 | 0.54 | 0.89 | 1.72 | 2.47 | 4.07 |
| 58 | -0.14 | 0.62 | 1.93 | 2.79 | 4.18 | 6.03 | 7.41 | 7.56 |
| 59 | -0.08 | 0.02 | 1.09 | 1.92 | 2.97 | 4.38 | 5.33 | 5.41 |
| 60 | -0.68 | 1.24 | 2.50 | 3.49 | 4.90 | 5.70 | 6.85 | 7.52 |

The table showed that reproducibility of identifying sTEA on No. 6 and No. 15 knee model was poor, while the reproducibility of No. 29 and No. 56 was relatively good.


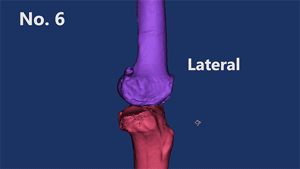


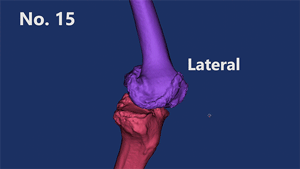


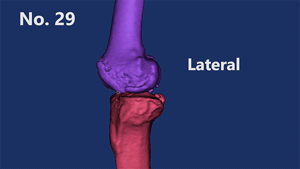


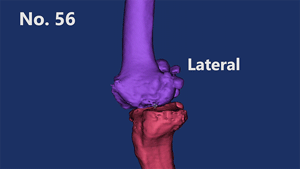


The above four dynamic graphics (if dynamic graphics is not moving, please check out videos in Appendix 2-5) showed 3D knee model of No. 6, 15, 29 and 56, respectively. It could be found that bony anatomical landmarks of No. 6 and No. 15 were obscurer than those of No. 29 and No. 56, especially in the medial femoral epicondyle, which indicates that identifiability of bony anatomical landmarks is positively correlated with the reproducibility of identifying sTEA.
